# Inclusion of the insecticide fenitrothion in dimethylated-β-cyclodextrin: unusual guest disorder in the solid state and efficient retardation of the hydrolysis rate of the complexed guest in alkaline solution

**DOI:** 10.3762/bjoc.9.14

**Published:** 2013-01-17

**Authors:** Dyanne L Cruickshank, Natalia M Rougier, Raquel V Vico, Susan A Bourne, Elba I Buján, Mino R Caira, Rita H de Rossi

**Affiliations:** 1 Department of Chemistry, University of Cape Town, Rondebosch 7701, South Africa; 2 Instituto de Investigaciones en Físico Química de Córdoba (INFIQC-CONICET), Departamento de Química Orgánica, Facultad de Ciencias Químicas, Universidad Nacional de Córdoba, Ciudad Universitaria, X5000HUA, Córdoba, Argentina

**Keywords:** crystal structure, cyclodextrin, fenitrothion, hydrolysis, inclusion complex

## Abstract

An anhydrous 1:1 crystalline inclusion complex between the organophosphorus insecticide fenitrothion [*O*,*O*-dimethyl *O*-(3-methyl-4-nitrophenyl)phosphorothioate] and the host compound heptakis(2,6-di-*O*-methyl)-β-cyclodextrin (DIMEB) was prepared and its structure elucidated by single-crystal X-ray diffraction. This revealed two independent host molecules in the asymmetric unit. In one of these, the cavity is occupied by two disordered guest components (distinguishable as rotamers with respect to the P–OAr bond) while in the other, three distinct guest components with site-occupancies 0.44, 0.29 and 0.27 appear, the last having a reversed orientation relative to all the other components. Kinetic studies of the alkaline hydrolysis of fenitrothion in the presence of DIMEB showed a remarkable reduction of 84% in the rate of this reaction relative to that for the free substrate, a value exceeding those previously attained with the native hosts, β- and γ-cyclodextrin, and fully methylated β-cyclodextrin.

## Introduction

Whereas cyclodextrins (CDs) have been employed for many years in the pharmaceutical industry to modify drug-delivery properties, the application of CD technology to the improvement of agrochemicals is a more recent innovation [1–2]. Nevertheless, very significant advantages of encapsulating agrochemicals such as pesticides (insecticides, herbicides, fungicides) in CDs may be gained [1], including, e.g., the conversion of toxic volatile liquids into solids, more localised pesticide application to improve delivery and reduce wastage, and stabilisation of the included pesticide against undesired degradation reactions.

**Figure 1:**
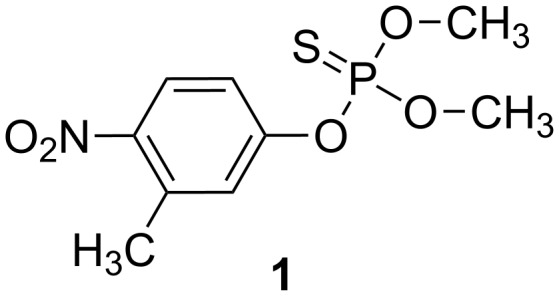
Chemical structure of fenitrothion (**1**).

Fenitrothion [*O*,*O*-dimethyl *O*-(3-methyl-4-nitrophenyl)phosphorothioate] (**1**, Figure 1) is an organophosphorus insecticide and acaricide [3]. It is effective against a wide range of pests that damage forests and various crops and it can also be used in the form of a residual contact spray to control flies, mosquitoes and cockroaches. Fenitrothion has also been employed in antimalarial programmes, where spraying of houses and animal shelters with this insecticide over extended periods significantly reduced the incidence and prevalence of the disease [4].

In our recent reports on the interaction between CDs and the organophosphorus insecticide **1** we gave an account of the X-ray crystal structures and thermal decomposition profiles of two solid inclusion complexes between permethylated α- and β-cyclodextrins, hexakis(2,3,6-tri-*O*-methyl)-α-CD (TRIMEA) and heptakis(2,3,6-tri-*O*-methyl)-β-CD (TRIMEB) and the guest [5]. We have also described the results of solution-based kinetic studies aimed at determining the effect of the presence of various CDs on the rate of alkaline hydrolysis of fenitrothion in aqueous solution containing 2% dioxane at 25 °C [6]. A crystalline inclusion complex (TRIMEA)_2_·**1** was isolated and shown by X-ray analysis to contain a novel capsule formed by head-to-head contact of the secondary rims of two TRIMEA molecules [5]. The encapsulated fenitrothion molecule showed minor disorder in the form of two rotamers (with respect to the P–OAr bond). In contrast, with the host TRIMEB, a monomeric inclusion complex TRIMEB·**1** was obtained, the guest molecule being statistically disordered over two positions [5]. In both crystalline complexes, however, the sensitive phosphate ester moiety was found to be buried deep within the CD cavity and we suggested that if this feature were to be obtained in solution, it would account for earlier observations that the rate of alkaline degradation of the ester is retarded in the presence of CDs [7–8].

Subsequently, a study of the kinetics of hydrolysis of the insecticide **1** in aqueous solution containing 2% dioxane at 25 °C and in the presence of the native cyclodextrins α-CD, β-CD, γ-CD as well as the permethylated derivatives TRIMEA and TRIMEB, was performed [6]. This revealed weak host–guest association in the case of α-CD and insoluble complex formation in the case of the host TRIMEA [9], whereas for β-CD, γ-CD and TRIMEB the association constants for the inclusion complexes of **1** had the respective values 417, 99 and 511 M^−1^ [6], the hydrolytic decomposition of **1** being significantly retarded by all three CDs.

The present study relates to the interaction between **1** and heptakis(2,6-di-*O*-methyl)-β-CD (DIMEB), the latter molecule having properties intermediate between those of the native and fully methylated counterparts [10]. The preparation and physicochemical characterization of the crystalline inclusion complex DIMEB·**1** are presented, this complex crystallizing in a novel arrangement and showing severe guest disorder, which was eventually resolved and successfully modelled. In addition, the results of a kinetic study of the hydrolytic degradation of **1** in the presence of DIMEB are reported and compared with those observed for the other CDs under the same reaction conditions.

## Results and Discussion

### Thermal analysis

**Figure 2:**
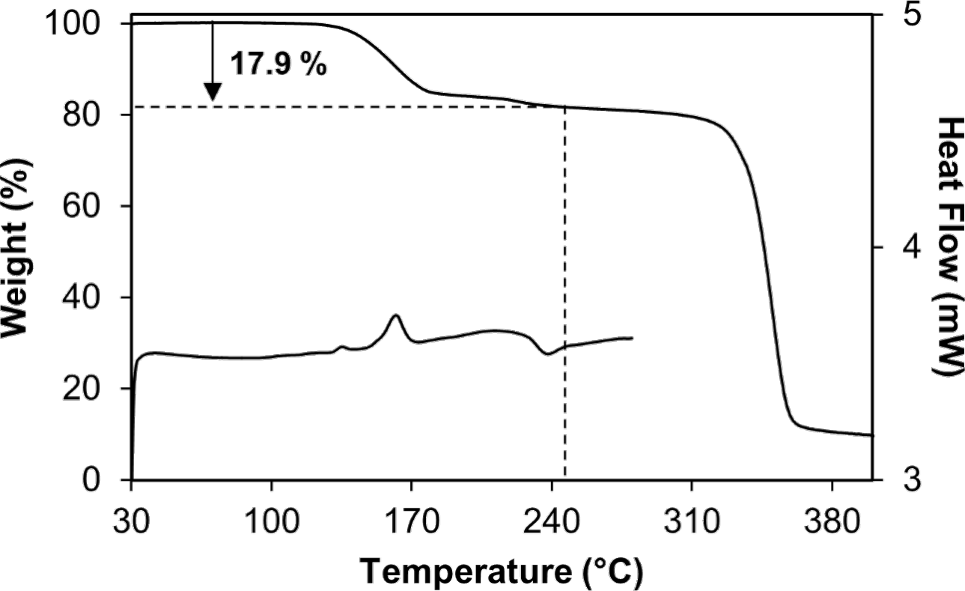
Representative TGA (top) and DSC (bottom) traces for DIMEB·**1**.

Figure 2 shows representative thermogravimetric (TGA) and differential scanning calorimetric (DSC) traces for the novel inclusion complex DIMEB·**1**. Three reproducible thermal events occur in the DSC trace, the first of which is a small endotherm at 132 °C, which we interpret as a minor solid–solid phase change, based on supporting hot stage microscopic (HSM) observations that show the initially colourless crystals turning opaque in this temperature region. A significantly larger endotherm follows at 158 °C accompanying the major phase of guest loss, while the broad exotherm at ~230 °C is interpreted as indicating the onset of host decomposition. The latter event is also confirmed by HSM showing the opaque crystals turning brown in the temperature region following guest loss. In the TG trace, major host decomposition is seen to occur above 300 °C.

Complete loss of the guest from the inclusion complex resulted in an estimated 17.9 ± 0.3% (*n* = 2) mass loss, which is in good agreement with the theoretical value of 17.2% for a 1:1 host–guest complex. The complex stoichiometry deduced from thermal analysis was subsequently confirmed by NMR spectroscopy, as described below.

### NMR spectroscopy

From the ^1^H NMR spectrum of the solid complex dissolved in CDCl_3_, a 1:1 DIMEB·**1** stoichiometry was calculated. Table 1 lists the relevant host and guest proton integrations, showing however, that a small excess of DIMEB, cocrystallized with the complex, was present in the sample.

**Table 1:** Integrals of the NMR signals for the protons of solid complex DIMEB·**1** dissolved in CDCl_3_ used to confirm the complex stoichiometry.

[Theoretical number of protons](DIMEB·**1**)	δ (ppm)	Experimental integration of peaks	Experimental peak integral/theoretical proton number

[7 × H1] (DIMEB)	4.952	9.02	1.3
[7 × H2] (DIMEB)	3.271	8.47	1.2
[3 × CH] (Ar-C*H*_3_, **1**)	2.621	3.00^a^	1

^a^Reference integral.

Fenitrothion is known to decompose at elevated temperature [11]. Since the complex crystals had been isolated at 60 °C, the possibility that the fenitrothion may have degraded thermally was checked by ^31^P NMR spectroscopy. Pure, uncomplexed fenitrothion dissolved in CDCl_3_ shows a single peak at 65.60 ppm. The complex crystals dissolved in CDCl_3_ also showed the presence of only one signal at 65.47 ppm confirming the structural integrity of the included insecticide molecule.

The ^31^P NMR spectrum of a solution of **1** (2 mM) in 2% dioxane-*d*_8_/D_2_O gave a single peak at 65.14 ppm, while in the same solution but in the presence of DIMEB (2 mM) the signal appears at 66.82 ppm, in agreement with data reported for **1** in water [6,12–14]. The shift observed indicates that a complex is formed.

The ^1^H NMR spectrum shows that the protons H3 and H5 of the cyclodextrin rings that are located inside the cavity are slightly shifted downfield, confirming the inclusion of the guest into the cavity.

### X-ray analysis

Table 2 lists the crystal data and data-collection parameters for the complex. No DIMEB-containing crystals with similar unit cell dimensions and the same space group as those of the DIMEB·**1** crystals were identified in the Cambridge Crystallographic Database [15], which necessitated its ab initio structural solution by using direct methods. Density considerations indicated an asymmetric unit containing two complex molecules. In addition, prior to structural solution, a special feature was evident from the X-ray diffraction pattern, namely the general alternation of strong and weak intensities for the reciprocal lattice levels *hkl* with *h* even and odd, respectively. This immediately indicated that the unit cell contents at *x*, *y*, *z* and *x +* 1/2*, y, z* are quite similar and that the correct direct-methods solution should be consistent with this prediction.

**Table 2:** Crystal data and data-collection parameters.

Compound	DIMEB·**1**

Chemical formula	C_56_H_98_O_35_·C_9_H_12_O_5_NPS
Formula weight / (g mol^−1^)	1608.57
Crystal system	Monoclinic
Space group	*P*2_1_
*A /* (Å)	19.626(2)
*B /* (Å)	15.049(2)
*C /* (Å)	26.854(3)
α / (°)	90
β / (°)	96.451(2)
γ / (°)	90
*V* / (Å^3^)	7881.0(15)
*Z*	4
*D*_c_* /* (Mg m^−3^)	1.356
μ[MoKα] (mm^−1^)	0.156
*F*(000)	3432
Temperature of data collection / (K)	173(2)
Crystal size / (mm)	0.46 × 0.35 × 0.26
Range scanned θ/ (°)	2.04–28.39
Index ranges ±*h*, ±*k*, ±*l*	−26, 26; −20, 19; −35, 35
Φ and ω scan angles / (°)	0.5
Total no. of frames	2494
Crystal to detector distance / (mm)	50.00
Total no. of reflections collected	125785
No. of independent reflections	20368
No. of reflections with *I >* 2σ*(I)*	17090
*R*_int_	0.0383
No. of refined parameters	1636
No. of least-squares restraints	75
Goodness-of-fit, *S*	1.019
R_1_ [*I >* 2σ*(I)*]	0.0770
No. of reflections omitted	34
*wR* on *F*^2^	0.2129
Weighting scheme parameters	a = 0.144 and b = 7.338
(Δ/σ)_mean_	<0.001
Δρ excursions (eÅ^−3^)	1.30 and −0.98
CCDC no.	898328

**Figure 3:**
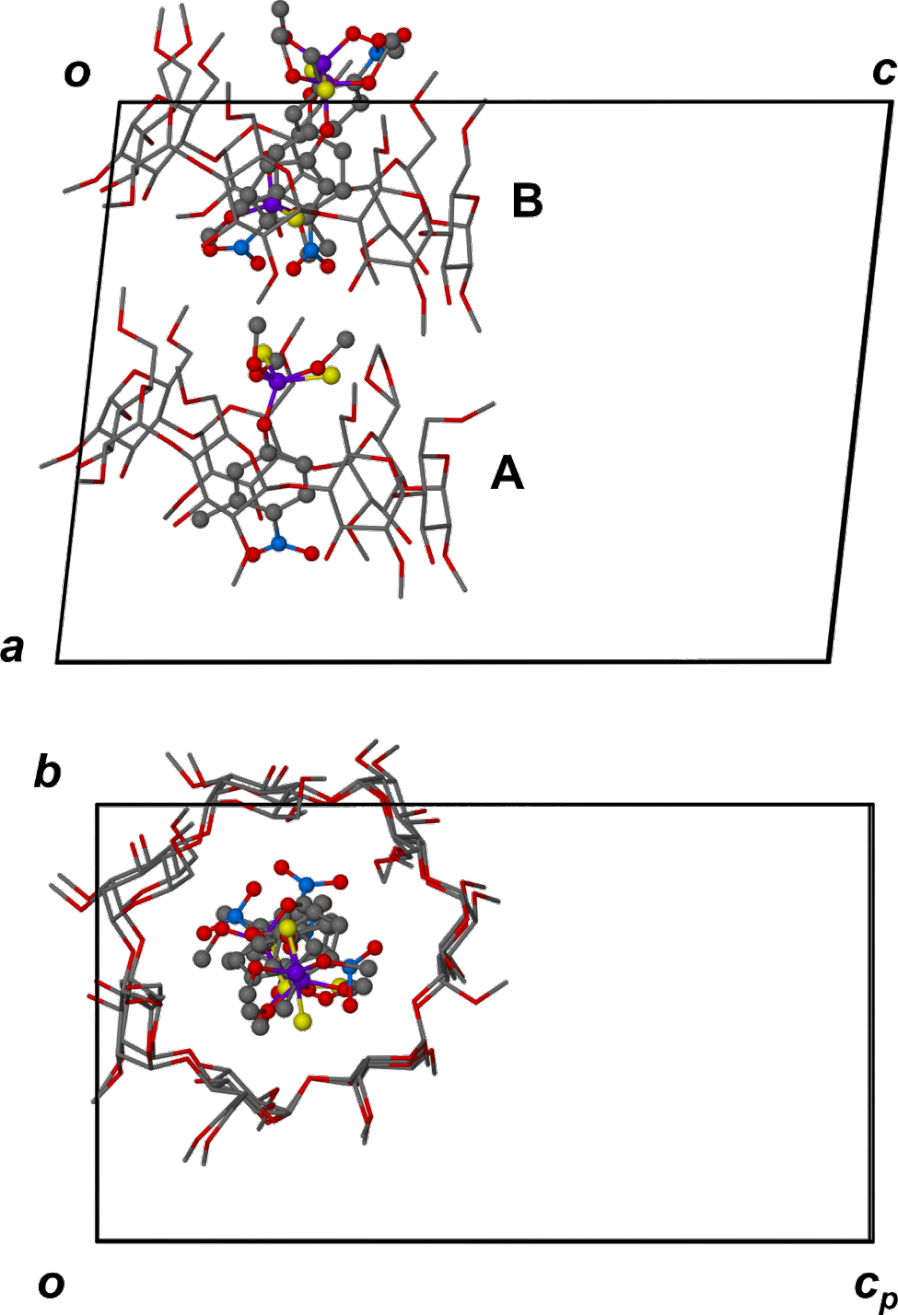
The asymmetric unit in DIMEB•**1** viewed along [010] (top) and [100] (bottom). H atoms are omitted for clarity.

This pseudo-translational symmetry was indeed a feature of the structural solution and is illustrated in Figure 3, where it relates the two complex units A and B in the crystal asymmetric unit of DIMEB·**1**.

**Figure 4:**
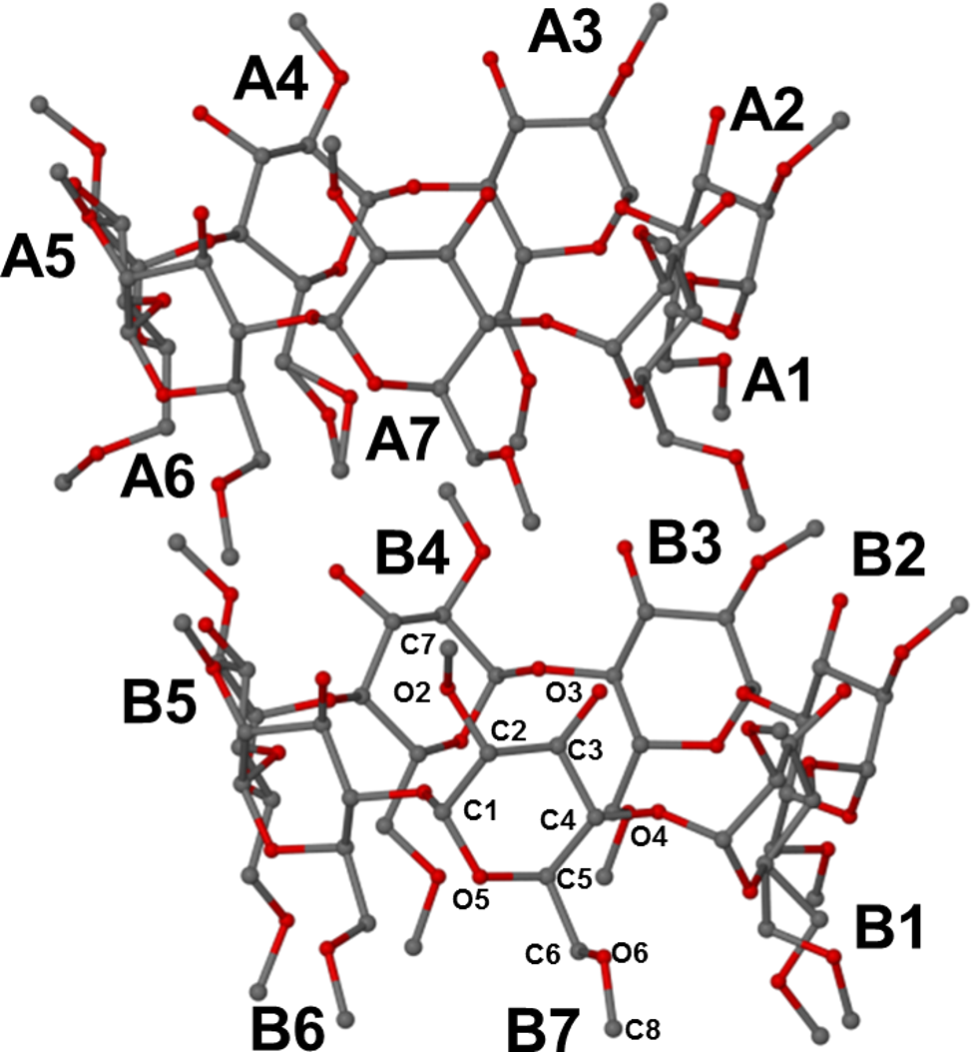
The host molecules in the asymmetric unit of DIMEB·**1** with the labelling of both residues and atoms indicated. H atoms are omitted for clarity.

While the gross structures of the independent complex units appear similar, there are obvious differences in the conformations of the two independent host molecules, and in particular, the images of the respective guest molecules are very different, as described further below. This is due to an unusually high level of guest disorder occurring in this complex, despite the low temperature of the data collection. Figure 4 shows the structures and atomic numbering of the two independent host molecule A and B, whose conformational parameters are listed in Table 3.

**Table 3:** Conformational parameters of the host molecules.^a^

*Res*	*l* (Å)	*D* (Å)	Φ (°)	*d* (°)	α (Å)	*D_3_* (Å)	τ_1_ (°)	τ_2_ (°)

A1	5.18	4.35	123.6	−11.0	−0.240	2.864	18.5	18.1
A2	4.74	4.51	135.9	0.3	0.122	2.849	14.7	19.7
A3	5.09	4.32	125.2	7.8	0.169	3.049	6.1	6.6
A4	5.20	4.34	125.7	0.4	−0.171	2.831	19.8	19.1
A5	4.97	4.40	128.7	−13.5	−0.134	2.744	15.9	17.0
A6	4.80	4.37	132.3	9.5	0.268	2.715	8.8	12.6
A7	5.11	4.22	126.8	5.5	−0.014	2.954	11.3	12.3

B1	5.31	4.42	121.1	−6.5	−0.078	2.991	15.5	16.5
B2	4.65	4.56	137.7	4.7	0.147	2.925	14.5	17.0
B3	5.03	4.23	127.4	3.3	0.018	2.906	13.9	13.4
B4	5.33	4.32	123.1	−2.6	−0.137	2.841	17.4	17.5
B5	4.97	4.47	129.4	−6.8	0.009	2.796	10.1	11.5
B6	4.72	4.42	134.0	10.7	0.174	2.755	9.9	11.9

B7	5.18	4.16	126.5	−2.5	−0.132	2.887	2.0	4.1

^a^The mean e.s.d.s for α, *D_3_*, τ_1_ and τ_2_ are 0.003 Å, 0.005 Å, 0.1° and 0.2° respectively.

The parameters in Table 3 that define the host structures include commonly employed descriptors of the O4-heptagon of each cyclodextrin molecule as well as the inclinations of the individual dimethylglucose residues (A1–A7, B1–B7, headed *Res* in Table 3) relative to the mean plane of the O4 atoms. These parameters are defined as follows: ***l***, the distance of each O4 atom from the centroid of the O4-polygon; *D*, the glycosidic O4···O4′ distance; Φ the O4···O4′···O4′′ angle; *d*, the O4···O4′···O4′′···O4′′′ torsion angle; *α*, the deviation of each O4 atom from the O4 mean plane; *D_3_*, the O2···O3′ intramolecular hydrogen bond distance; tilt angle τ_1_, the angle between the six atoms of the glucopyranose ring (C1, C2, C3, C4, C5, O5) and the line orthogonal to the O4 mean plane; tilt angle τ_2_ between the O4 plane and the mean plane through atoms O4, C4, C1 and O4′ of a given glucose ring.

All the dimethylglucose rings of host molecules A and B adopt the ^4^*C*_1_ chair conformation. On their primary sides, with few exceptions, the values of the torsion angles ω (O5–C5–C6–O6) indicate (−)-*gauche* conformations, with the C6–O6 bonds thus directed away from the centres of the respective cavities. This results in relatively open cavities, contrary to the usual situation in inclusion complexes of the fully methylated host analogue TRIMEB, where the primary methoxy groups typically act as a “lid”, sealing that side of the macrocycle. In the DIMEB·**1** complex, there is thus no distinct “boundary” separating the guest molecules within the cavity of host molecule A from those within host molecule B.

The tilt angles (τ_1_ and τ_2_) are all positive indicating that each ring has its primary side tipped towards the centre of the cavity. The slight ellipticity in the host conformations, reflected in the values of *l, D* and Φ (Table 3) is a compromise between the tendency for “roundness” (induced by the intramolecular O2···O3′ hydrogen bonds with lengths *D_3_*) and slight distortion due to guest inclusion. The O4 mean planes of host molecules A and B are slightly offset from one another but are nearly parallel (interplanar angle 2.65(2)°).

**Figure 5:**
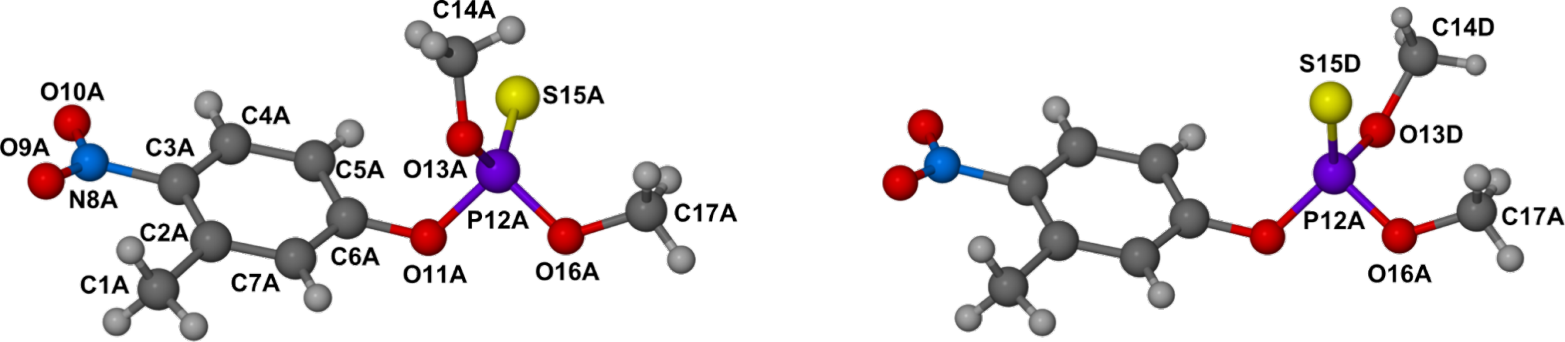
The rotamers of **1** occupying the cavity of host molecule A. Common atoms have labels with suffix A, while in the component on the right the alternative sulfur and methoxy group positions are labelled with suffix D. These disordered guest components (labelled A, D) have site occupancies 0.70 and 0.30, respectively.

Details of the guest disorder within the host A cavity are illustrated in Figure 5.

Here, a single molecule of fenitrothion occupies the cavity, being present as two rotamers whose phosphorothioate groups are related by a rotation of 126° around the O11–P12 bond. The phosphorothioate group of each disordered component of the guest **1** is located near the primary side of the host molecule.

**Figure 6:**
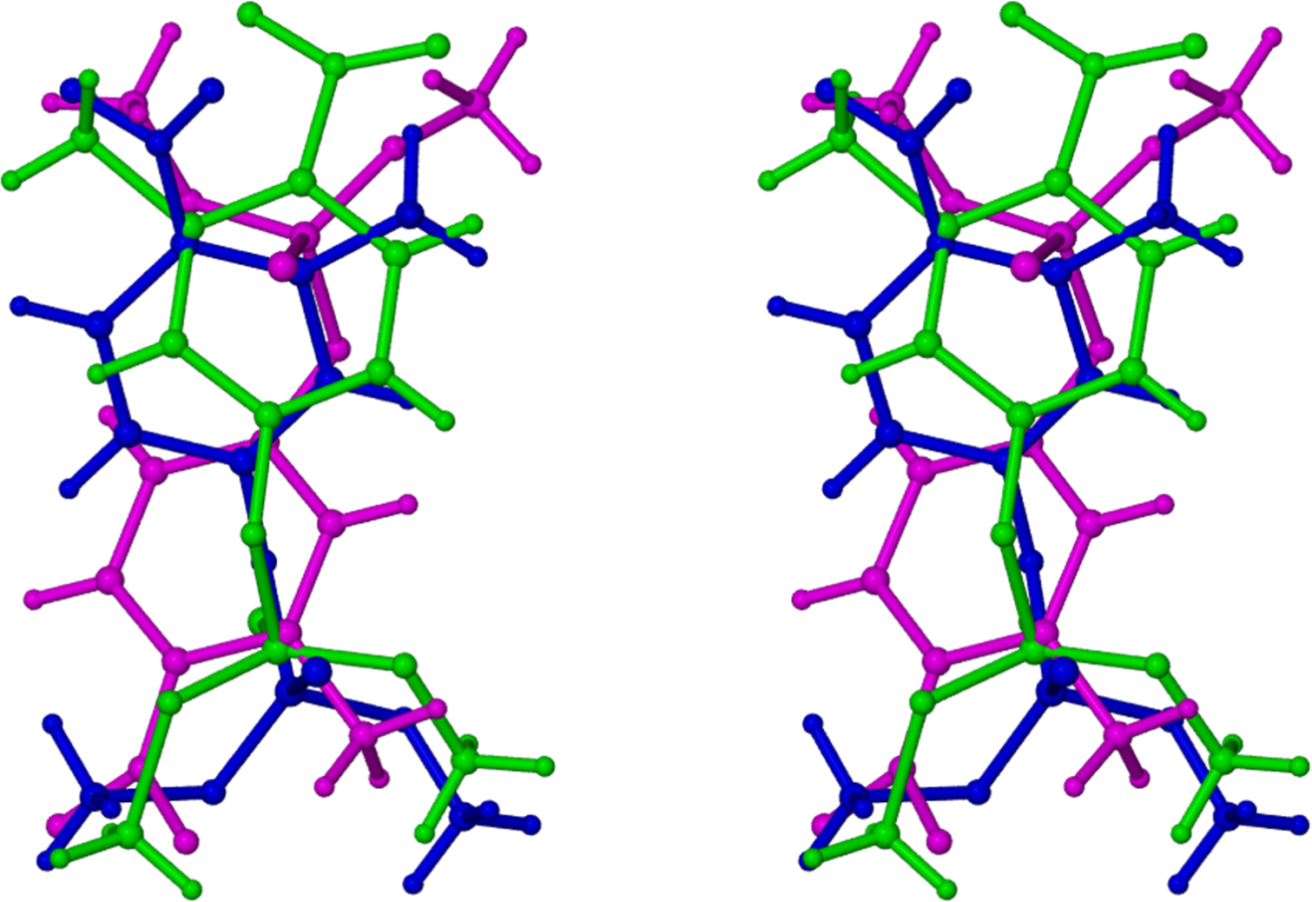
Stereoview of the three disordered guest components that occupy the cavity of host molecule B. Guest components B (green) and C (blue) have similar orientations while component E (pink) adopts the opposite orientation.

Instead, within the cavity of host molecule B, as many as three disordered guest components (B, C, E) were observed and modelled, as shown in Figure 6. These disordered components (B, C, E) are shown in green, blue and pink, respectively, in the stereoview of Figure 6.

The major disordered guest components B and C, with respective site-occupancy factors (sofs) 0.44 and 0.29, adopt the same orientation, their phosphorothioate groups being located near the primary side of host molecule B (analogous to the situation in host molecule A). However, the third guest component E, with the lowest sof (0.27), adopts the opposite orientation to those of components B and C. This low sof indicates that the guest orientations adopted by the other four disordered components (A–D) is the preferred one in the solid state.

Host molecules A and B have slightly different geometries (e.g., extents of ellipticity), as indicated by the parameters in Table 3. More detailed analysis shows that these differences are attributable to their accommodation of different assemblies of disordered guest components.

An analysis of hydrogen bonding in the crystal of DIMEB·**1** revealed several types of interactions. These can be summarised as follows: (a) For the two independent host molecules A and B, a total of 14 intramolecular H-bonds O2*n···*H–O3(*n*−1) characteristic of the DIMEB molecule (O···O distances in the range 2.715(5)–3.049(5) Å), linking contiguous glucose residues and thus inducing a “round” host conformation (parameter *D_3_* in Table 3); (b) intramolecular H-bonds C6–H6···O5′/O6′ and C7–H7···O3′, which also contribute to stabilisation of the host conformation; and (c) 10 unique host–host intermolecular C–H···O hydrogen bonds that stabilise the host framework.

**Figure 7:**
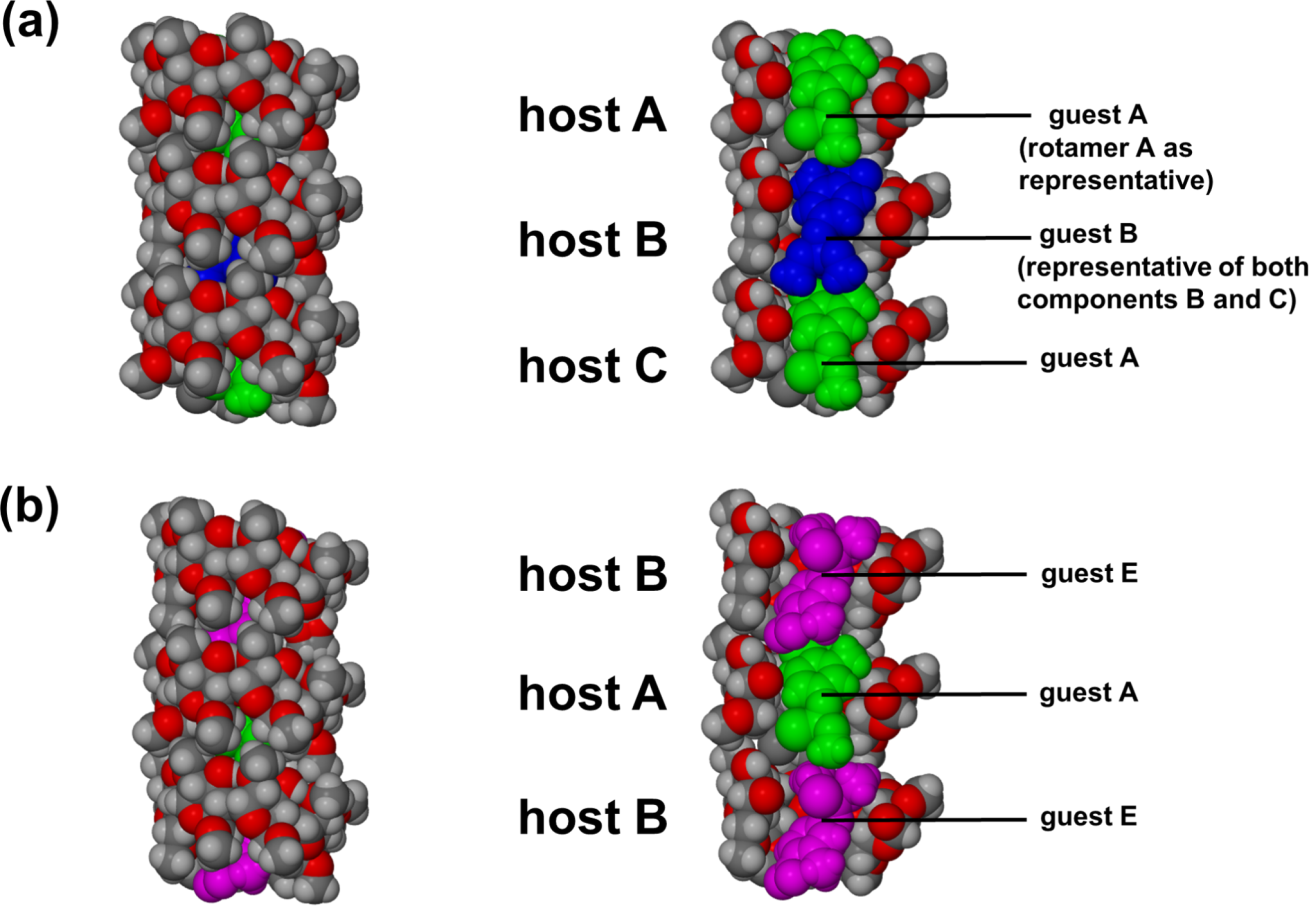
Space-filling diagrams showing the relative orientations of guest molecules within the cavities of the independent DIMEB molecules. Side-views of the complexes and cross-sectional views showing guest molecules (a) A and B, and (b) guest molecules A and E, encapsulated by their respective host molecules.

All guest molecules are fully encapsulated by the host molecules and there are no close contacts between the disordered guest molecules and host molecules. Figure 7 shows space-filling diagrams that illustrate representative modes of guest inclusion.

**Figure 8:**
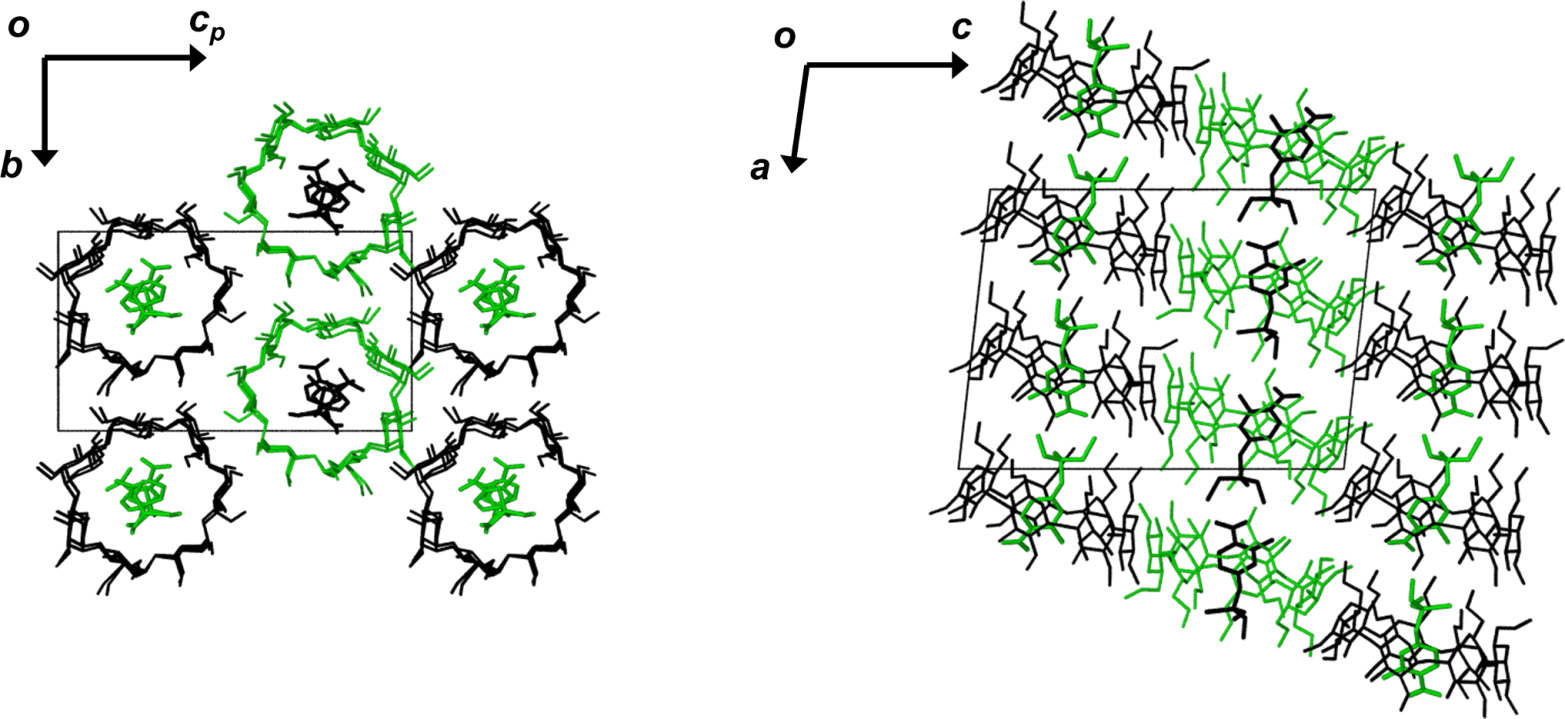
Packing diagrams of the DIMEB·**1** structure, viewed along [100] (left) and [010] (right). The symmetry related molecules are distinguished by the reverse green and black host and guest molecules. For clarity, only guest molecules A and B are shown and the hydrogen atoms have been omitted.

Crystal packing is shown in Figure 8. The complex units stack head-to-tail in infinite columns parallel to the *x*-direction. Owing to the twofold screw axis parallel to the *b*-axis, the adjacent columns are antiparallel. The slight ellipticity of the host molecules and the extension of the primary methoxy groups into the interstitial sites assist in the close packing of the complex units.

### UV–vis and ICD studies

Spectroscopic methods are frequently used to determine association constants between cyclodextrins and different guests [16]. We attempted to determine the binding constant of **1** with DIMEB by UV–vis spectroscopy; however, when we recorded the spectra of **1** in the presence of DIMEB, only a small hypsochromic shift was noted and the change in absorbance was not large enough for an accurate determination of the binding constant.

**Figure 9:**
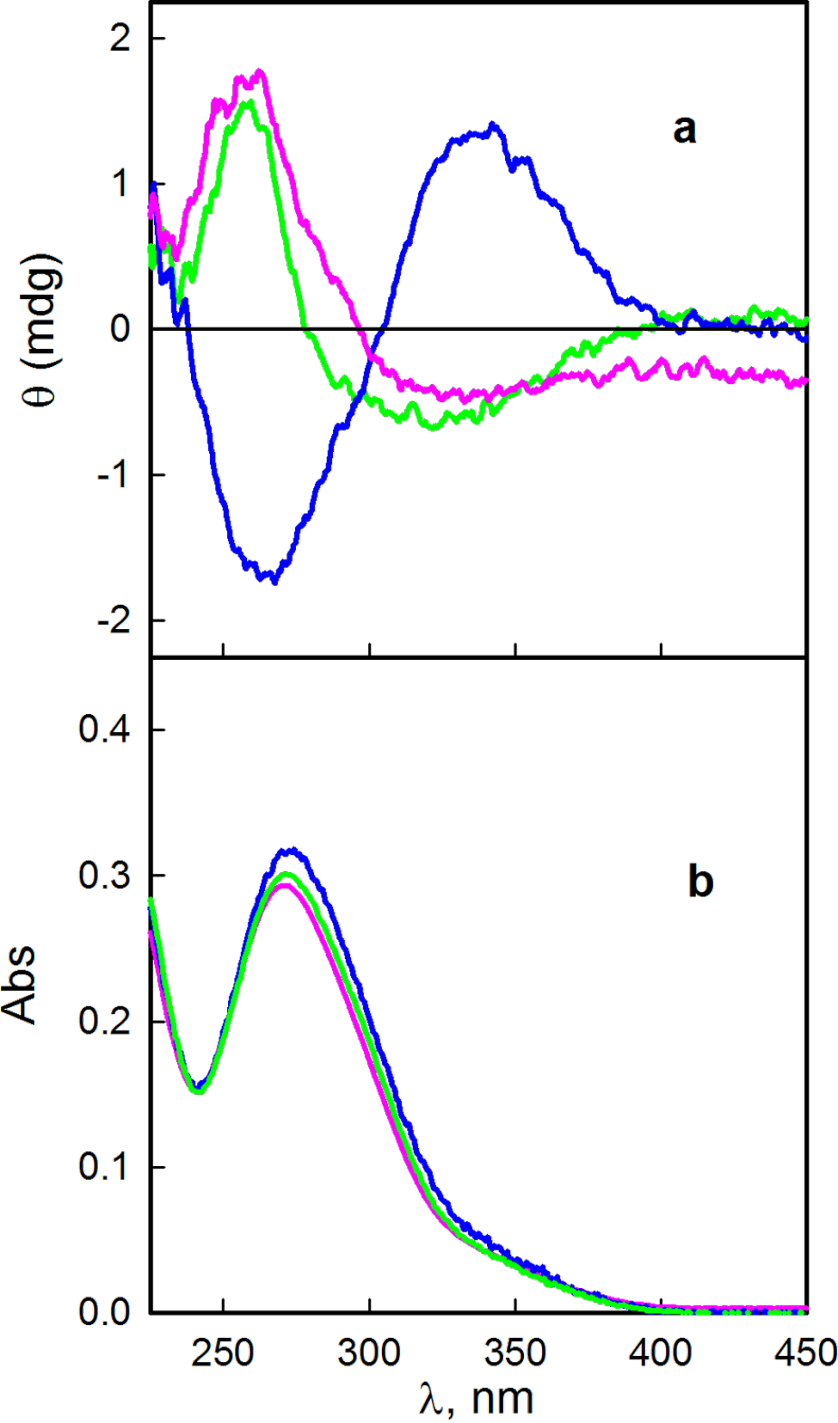
Induced circular dichroism (a) and UV–vis (b) spectra of **1** in the presence of β-CD (10 mM, green), DIMEB (2.5 mM, pink) and TRIMEB (10 mM, blue).

The induced circular dichroism (ICD) spectrum of DIMEB and **1** is shown in Figure 9a, where the spectrum recorded for β-CD and TRIMEB with **1** is also shown for comparison.

It can be seen that the native β-CD and DIMEB show a negative and a positive peak (Figure 9a). The negative peaks appear at 320 and 324 nm and the positive ones at 260 and 265 nm for β-CD and DIMEB, respectively. The positive peak in the circular dichroism spectrum is slightly displaced from the maximum of the UV–vis spectrum as was observed in other cases where cyclodextrin was the chiral component [17–20].

The ICD spectrum of fenitrothion in the 200–300 nm wavelength range in the presence of β-CD was reported in the literature [21] and the spectrum shows a negative peak at ≈220 nm and a positive peak at ≈280 nm. This spectrum was measured for a basic solution and in the paper it is not stated what time had elapsed between the preparation of the solution and the running of the spectrum. We suspect that the spectrum reported corresponds to that of the hydrolysis product, namely 3-methyl-4-nitrophenol **2**. We carried out measurement of the ICD spectrum of **2** in a 3% dioxane–water solution, and the spectrum obtained was similar to that reported by Kamiya et al. [21] for fenitrothion (**1**).

It is remarkable that the sign of the peaks in the ICD spectrum of **1** in the presence of TRIMEB is inverted when compared with the spectra of β-CD and DIMEB with **1**. As suggested previously [6,8,22], the change in the sign of an ICD spectrum may indicate extrusion of the guest or a change in orientation of the guest in the cavity.

There are two possible modes of inclusion of the insecticide molecule in the cavity of a CD, either with the phosphorothioate moiety (type A) or with the aromatic moiety (type B) inside the cavity [6]. For most of the esters having an aromatic group it is proposed that this residue is included in the cavity of the CDs. This was suggested for parathion, methyl parathion, and paraoxon [17,21,23]. Theoretical calculation of the energies of the two types of complexes in the gas phase indicates that there is no significant difference in their values [24]. Single-crystal X-ray analysis shows that for β-CD and TRIMEB, complexes of type A are formed while for DIMEB (see above) in the unit cell a small percentage of the inclusion complexes exhibit the type B orientation.

By comparing the ICD spectra of the three complexes it seems that, in solution, the fenitrothion molecule adopts the same orientation in the β-CD and DIMEB cavities, which is different from that in the complex with TRIMEB. It appears that the presence of OH in the rim of the cavity has an important role in determining the orientation of the included guest molecule.

Based on the fact that the ICD spectra of *p*-nitrophenol and fenitrothion are similar, Kamiya [21] suggested that the inclusion of both compounds should be similar. This is not the case under our reaction conditions; therefore we think that the type A complex is predominant for β-CD and DIMEB.

### Kinetic studies

**Scheme 1:**
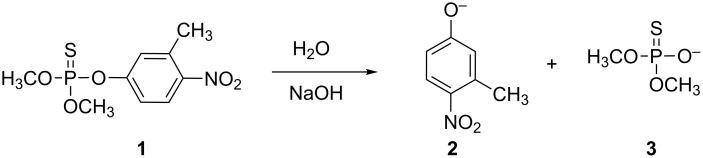
Reaction of fenitrothion in basic media.

The hydrolysis reaction of fenitrothion takes place with P–O bond fission as shown in Scheme 1 [12] and it was studied in the presence of constant HO^−^ and variable DIMEB concentrations, as reported previously with other CDs [6].

**Figure 10:**
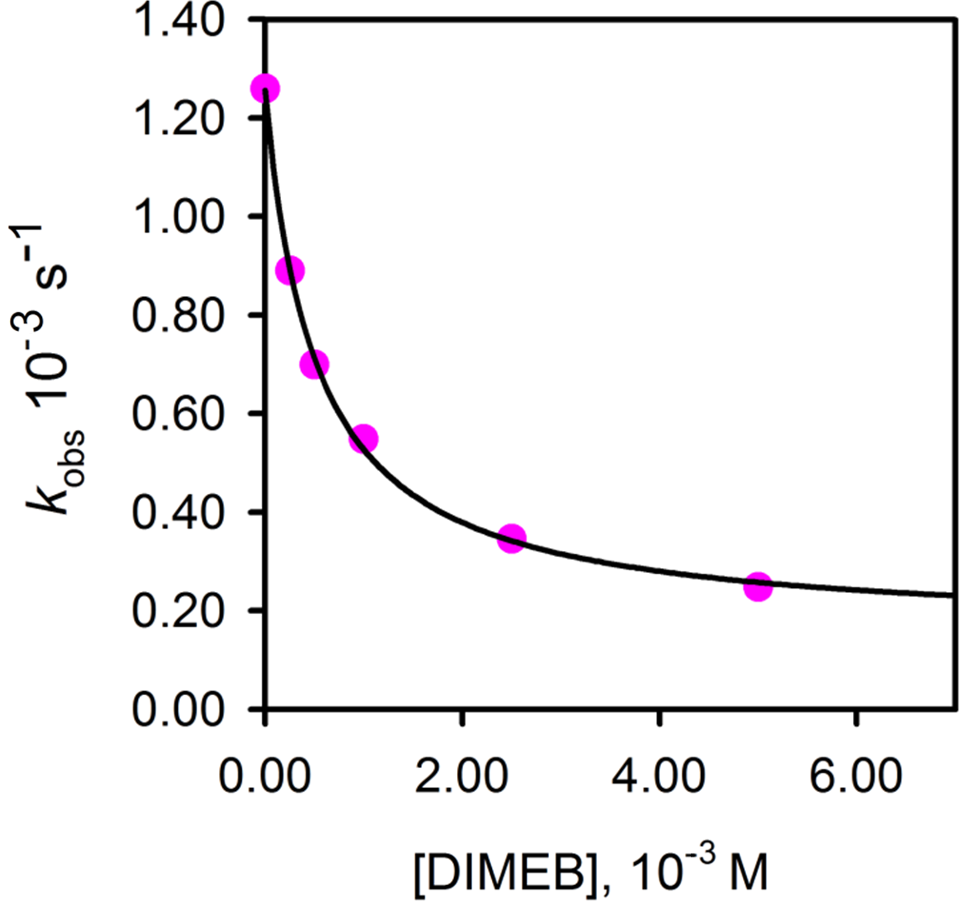
Plot of *k*_obs_ versus [DIMEB] for the hydrolysis reaction of fenitrothion with HO^–^ at different concentrations of DIMEB. *T* = 25 °C, [NaOH] = 0.5 M, ionic strength = 1 M (NaCl). Solvent: water with 2% 1,4-dioxane. The line was drawn by using the data calculated for *k*_0_, *k*_2_ and *K*_2_ in Equation 1.

In Figure 10 a plot of the observed pseudo-first-order rate constants *k*_obs_ as a function of the concentration of DIMEB is shown. The highest concentration of DIMEB used was 0.005 M. At a cyclodextrin concentration higher than this, precipitation of the DIMEB occurred under the conditions of our studies (ionic strength 1 M, NaOH 0.5 M aqueous solution with 2% 1,4-dioxane). This was confirmed by performing an experiment with DIMEB at a concentration of 0.01 M without the addition of substrate; a day later there was a solid precipitate at the bottom of the flask, which was confirmed by ^1^H NMR spectroscopy to be DIMEB.

**Scheme 2:**
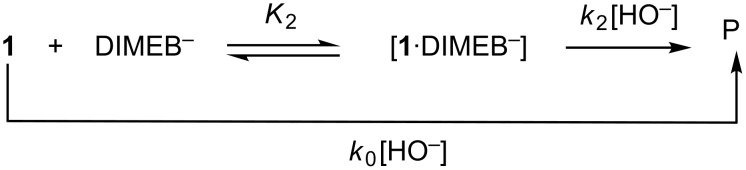
Mechanism of the hydrolysis reaction of **1** mediated by DIMEB.

The reaction of **1** with NaOH in the presence of DIMEB may take place as shown in Scheme 2 [7–8], where *k*_0_ and *k*_2_ represent the reactions of the free substrate and of the substrate complexed with ionized cyclodextrin (DIMEB^–^), respectively [25]. The observed rate constant for Scheme 2 is shown in Equation 1.

[1]
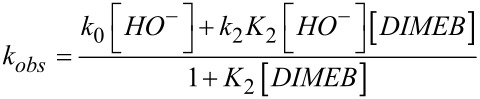


The values of the rate and equilibrium constants were obtained by treating the kinetic data as previously published [6] and they are (1.20 ± 0.01) × 10^–3^ s^–1^, (0.24 ± 0.04) M^–1^ s^–1^ and (1.69 ± 0.09) × 10^3^ M^–1^, for *k*_0_[HO^–^], *k*_2_*K*_2_[HO^–^], and *K*_2_, respectively. From these data *k*_2_ = 0.14 × 10^–3^ s^–1^ can be obtained and this is a measure of the rate of the reaction taking place within the DIMEB cavity.

The association constant determined for DIMEB and **1**, viz. (1.69 ± 0.9) × 10^3^ M^–1^ is significantly higher than the values previously measured for complexation of **1** with β-CD and TRIMEB, namely 417 and 511 M^–1^ [6]. Furthermore, the inhibition effect as measured by the ratio *k*_0_/*k*_2_ is also significantly higher, namely 7.5, 8.6 and 5.0 for TRIMEB, DIMEB and β-CD, respectively, indicating that DIMEB is more effective in terms of protecting the substrate against the reaction with the nucleophile. The value of *K*_2_ obtained by other authors under similar but not equal reaction conditions is 189 M^–1^ [21]. Part of the discrepancy with our results can be attributed to the different reaction conditions, but we think that the main difference may come from the sample of DIMEB used in each work. It is remarkable that the kinetic data obtained for β-CD are in much better agreement.

The large difference in the inhibitory effect when compared to the native CDs may be due to the methyl groups present on DIMEB and TRIMEB, which elongate the cavity of the CD molecules and provide a slightly larger pocket in which the fenitrothion molecules can situate themselves. Furthermore, the methyl groups have the ability to block the entering hydroxide ions, thereby preventing nucleophilic attack at the phosphorus atom. From the X-ray crystal structures of the CD complexes of fenitrothion with TRIMEB [6] and DIMEB (see above), it is clear that the major components of the fenitrothion molecules have their reactive phosphorothioate moieties positioned at the narrower primary rims of the cyclodextrin cavities. The stronger inhibition of fenitrothion hydrolysis in the presence of DIMEB in comparison with TRIMEB may indicate that the phosphorothioate unit is more protected from nucleophilic attack when it is in the cavity of DIMEB. A DIMEB molecule has a more rounded shape when compared to TRIMEB, due to the intramolecular O2···H–O3' hydrogen bonds that link neighbouring methylglucose residues. As a result, there is more available space in the cavity of a DIMEB molecule than in that of a TRIMEB molecule, and therefore the guest fenitrothion can penetrate further into the cavity.

## Conclusion

Single-crystal X-ray analysis of the 1:1 DIMEB fenitrothion inclusion complex revealed that the mode of inclusion of the disordered guest molecules is predominantly of type A (phosphorothioate group near the host primary side). ICD spectra of the DIMEB and β-CD complexes indicated that analogous type A inclusion prevails in solution. The significantly higher association constant reported here for fenitrothion complexation with DIMEB in aqueous solution compared with previous data for its complexation with β-CD and TRIMEB results in more effective protection of the insecticide molecule from hydrolytic degradation.

Kinetic data of the type established in this and previous studies that we have undertaken, give indications of which type of cyclodextrin may be best suited in an agrochemical formulation to ensure resistance of sensitive bioactive compounds to chemical degradation, thus maximising their intended biological effect. Thermodynamic data, such as complex association constants, are also relevant parameters, especially in the context of CD-mediated remedial treatment of soils that are contaminated by persistent pesticides. As regards fenitrothion, a peripheral but important advantage of its inclusion in CDs is the significant reduction in its volatility (and hence toxicity) by conversion of the liquid pesticide into a solid phase.

## Experimental

The compounds used in this study were purified as indicated before [6].

### Crystal preparation and X-ray diffraction analysis

Fenitrothion (**1**, 20 mg, ~0.072 mmol) was added to a saturated aqueous solution of DIMEB (96 mg, 0.072 mmol) at 20 °C. Continuous stirring for 6 h, as well as several heating and cooling cycles between 20 and 60 °C led to formation of a clear solution, which was then filtered into a clean vial, capped and placed in an oven at 60 °C. Colourless single crystals of the complex DIMEB·**1** appeared over a 24 h period.

Collection of intensity data was performed on a Bruker KAPPA APEX II DUO diffractometer with the crystal cooled in a constant stream of nitrogen vapour (Oxford Cryostream, UK). Upon inspection of the reciprocal lattice layers with the program LAYER [26], the X-ray diffraction pattern revealed Laue symmetry 2/*m*, corresponding to the monoclinic crystal system. The intensity-weighted reciprocal lattice also revealed alternation in intensity of the layer lines perpendicular to a*, from which it was deduced that the structural motif at *x*, *y*, *z* repeats itself at approximately *x + ½, y, z*. The space group *P*2_1_ was identified from the reflection conditions *hkl*: none; *h*0*l*: none; 0*k*0: *k =* 2*n* (the alternative, centric space group *P*2_1_/m was eliminated since it could not accommodate the chiral cyclodextrin host). Owing to the alternating strong and weak reflections it was possible to deduce the presence of two DIMEB molecules in the asymmetric unit.

As no isostructural DIMEB complex could be found in the literature, the program SHELXD [27] was employed to solve the structure by ab initio direct methods. The initial structural solution (correlation coefficient = 84.9) revealed most of the nonhydrogen host atoms of the two DIMEB molecules and a partial guest molecule in one of the DIMEB host cavities. Before the first refinement in SHELXH-97 [28] the carbon and oxygen atoms of the host were correctly assigned, as were the atoms of the first guest molecule. The remaining host atoms were located in the initial and subsequent difference Fourier maps. The methyl glucose moieties of two independent CD molecules in the asymmetric unit were labelled A1–A7 and B1–B7.

One primary methoxy group per host molecule was disordered over two positions (on glucose residues B1 and A4). In the case of the primary methoxy group on methyl glucose unit B1, all three atoms (C6, O6 and C8) were disordered and the major component refined with a sof of 0.53, while methyl glucose unit A4 contained only atom O6, which was disordered over two positions (sof = 0.69) while C8 was shared. All ordered host atoms were refined anisotropically, except atoms C7A3, C7A7, C8B4 and C8B5, which had reasonable isotropic thermal parameters but unacceptable thermal ellipsoids when refined anisotropically.

The host hydrogen atoms were placed by using a riding model and were refined isotropically with thermal displacement parameters 1.2 times those of their parent atoms. For the hydroxy groups on each methyl glucose unit a hydrogen-bond searching model (AFIX 83) was used to place the hydrogen atoms in geometrically reasonable hydrogen-bonding positions. However, this procedure was not suitable for all the hydroxy groups, and thus, distance restraints were applied between the hydrogen atom of the hydroxy group and the hydrogen-bond-acceptor oxygen atom on the adjacent glucopyranose unit. This was necessary for hydroxy groups on methyl glucose units A4, A5, A6, A7, B6 and B1.

When both host molecules had been located, the first guest molecule that resulted from the initial SHELXD solution was modelled, followed by another in the second crystallographically independent DIMEB molecule. After refinement, the thermal displacement parameters of the guest in host molecule A were reasonable, with the exception of those of the atoms belonging to the *O,O*-dimethyl phosphorothioate moiety. The two disordered guest molecules were found to be rotamers that result from rotation of substituents around the O11–P12 bond. Distance restraints were applied to atoms of the phosphorothioate moiety and an AFIX 66 instruction was applied to the phenyl group, which constrained the ring as a rigid hexagon. The common atoms of the guest molecule were labelled A while the second sulfur and methoxy group were labelled D. The refined values of the sofs of the two rotamers were 0.70 and 0.30. All the ordered atoms of guest molecule A were refined anisotropically, with the exception of atoms O9A, O10A and C17A.

The first disordered guest component molecule modelled in host molecule B had many additional high electron-density peaks surrounding it and the entire molecule had unacceptably high thermal displacement parameters. There was an abnormally large peak situated close to the phenyl ring but it was not in the plane of the phenyl group. Two additional hexagonal rings were discerned in the difference Fourier map, suggesting two other guest-molecule positions. After many attempts at modelling the disorder, three disordered components within host molecule B were evident. The relative population of each was unknown and required an analysis of the initial electron-density peak heights for the phosphorus atoms of each component. Guest components B, C and E had initial phosphorus electron-density peaks of 4.66, 3.07 and 2.86 eÅ^−3^, respectively, which resulted in fixed sof values of 0.44, 0.29 and 0.27 being applied to the three disordered components. Owing to the excessive and unusual type of disorder of the guests present in this particular structure, many distance restraints and three AFIX 66 commands were applied to maintain reasonable geometries. Global isotropic thermal displacement parameters were assigned to each component and these refined to values of 0.10, 0.07 and 0.13 Å^2^ for guest molecules B, C and E, respectively. The guest hydrogen atoms were placed in idealised positions in a riding model with isotropic thermal displacement parameters in the range 1.2–1.5 times those of their parent atoms.

CCDC 898328 contains the supplementary crystallographic data for this paper. These data can be obtained free of charge at http://www.ccdc.cam.ac.uk/products/csd/request/ [or from the Cambridge Crystallographic Data Centre (CCDC), 12 Union Road, Cambridge CB2 1EZ, UK; fax: +44(0)1223-336033; email: deposit@ccdc.cam.ac.uk].

### Thermal analysis

Mass loss on heating the crystals of DIMEB·**1** was measured on a TA-Q500 Thermogravimetric Analyzer, TA instruments, equipped with Universal Analysis 2000 software. The differential scanning calorimetric trace was recorded on a DSC-Q200 calorimeter, TA instruments. For both techniques, a heating rate of 10 K min^−1^ and dry nitrogen purge gas flowing at a rate of 30 mL min^−1^ were employed. Crystals were transferred to an open alumina or aluminium crucible in the case of TG measurements and closed vented aluminium pans for DSC measurements. Sample masses were between 0.5 and 2.0 mg for DSC measurements and between 2 and 8 mg for the TG runs.

### Kinetic procedures

The kinetic procedures were described previously [6].

The DIMEB molecule has hydroxy groups that can be deprotonated by hydroxide ions in basic media; therefore, when calculating the NaOH concentration required for preparation of the solutions it is necessary to take this fact into account. Since we did not find values for the p*K*_a_ of DIMEB in the literature, we used the p*K*_a_ value of β-CD, viz. 12.20 [29–30].
